# Efficacy of osimertinib for preventing leptomeningeal metastasis derived from advanced EGFR-mutated non-small cell lung cancer: a propensity-matched retrospective study

**DOI:** 10.1186/s12885-021-08581-2

**Published:** 2021-07-30

**Authors:** Xia Wang, Jing Cai, Zhimin Zeng, Anwen Liu

**Affiliations:** 1 Department of Oncology, The Second Affiliated Hospital of Nanchang University, No.1 Minde Street, Nanchang, 330000 Jiangxi Province People’s Republic of China; 2 Jiangxi Key Laboratory of Clinical Translational Cancer Research, Nanchang, 330000 Jiangxi Province People’s Republic of China

**Keywords:** Non-small-cell lung cancer, Epidermal growth factor receptor, Tyrosine kinase inhibitors, Leptomeningeal metastasis, Osimertinib, Propensity score matching

## Abstract

**Background:**

Leptomeningeal metastasis (LM) is a severe complication of advanced non-small cell lung cancer (NSCLC). This retrospective study aimed to investigate the potential use of osimertinib for preventing LM in patients with advanced epidermal growth factor receptor (EGFR)-mutated NSCLC.

**Methods:**

Patients with advanced NSCLC harboring EGFR mutations who underwent tyrosine kinase inhibitors (TKIs) therapy for at least 8 weeks between September 2016 and September 2019 were eligible for this study. All included patients were divided into two groups based on whether they received osimertinib, the osimertinib group (patients treated with osimertinib) and the control group (patients not treated with osimertinib). Propensity score matching (PSM, ratio of 1:1) was used to account for differences in baseline characteristics. The cumulative incidence of LM and the overall survival (OS) were evaluated.

**Results:**

A total of 304 patients were included in the study population. Among them, 116 patients received osimertinib, and 188 did not. A total of 112 patients remained in each group after PSM, and the baseline characteristics were not significantly different between the two cohorts. LM developed in 11 patients (9.82%) in the osimertinib group and 24 patients (21.42%) in the control group (hazard ratio [HR] 0.38, 95% confidence interval [CI] 0.19–0.79, *p* = 0.009). Multivariate analysis indicated that osimertinib was an independent, statistically significant predictor for determining the risk for LM, with an HR of 0.33 (*p* = 0.042). At present, the OS rate data are too immature for statistical analysis.

**Conclusion:**

Real-world data demonstrate that osimertinib can significantly reduce the incidence of LM in patients with advanced NSCLC harboring common EGFR mutations. Given this result, osimertinib should be encouraged in clinical practice for specific patient populations.

## Background

Leptomeningeal metastasis (LM) is the seeding of malignant cells within the subarachnoid space and leptomeninges (pia and arachnoid mater) [1]. LM is a devastating complication of advanced non-small cell lung cancer (NSCLC) and typically occurs in 3–5% of patients [2, 3]. However, its incidence is higher in subgroups of patients with targetable molecular driver mutations and occurs in 9.4% of patients with epidermal growth factor receptor (EGFR) mutations, largely due to the improved overall survival (OS) obtained from new molecular therapies [3, 4]. The median OS of NSCLC patients with LM remains poor, though the OS has improved from the historical 1–3 months to 3–11 months as a result of novel therapies and the integration of local and systemic treatments [1, 5]. The clinical presentation of LM can include cranial nerve deficits, cauda equina symptoms or signs, visual disturbances, diplopia, hearing loss, neurocognitive syndromes and signs related to intracranial pressure in the later stages (headache, nausea/vomiting, gait difficulties, encephalopathy and somnolence). These bothersome symptoms often considerably impair the quality of life of patients with LM [6, 7].

Due to the existence of blood-brain barrier (BBB) and the low penetration of therapies into the cerebrospinal fluid (CSF), traditional therapies are often considered futile for preventing and treating brain metastasis (BM) and LM [1, 8, 9]. However, recent molecular targets have shown high penetration of the BBB and improved clinical outcomes for subgroups of patients with EGFR-mutated (EGFRm) NSCLC and LM [1, 3, 9–12]. A retrospective study showed that patients who received EGFR tyrosine kinase inhibitors (TKIs) had a longer OS time than those who did not (10.0 months vs 3.3 months, *p* < 0.001) [3]. Another study also revealed that EGFR-TKIs therapy after the diagnosis of LM was an independent predictor of extended survival in patients with EGFRm NSCLC [10]. Osimertinib, a third-generation TKI, has greater penetration of the BBB and higher brain infiltration capacity than first- and second-generation TKIs (erlotinib, gefitinib or afatinib) [1, 9, 13, 14]. In the AURA2 study, 22 patients with the EGFR T790M mutation and LM received osimertinib after their disease progressed during prior treatment TKIs, and had a median OS time of 18.8 months [11]. The National Comprehensive Cancer Network now recommends osimertinib for the treatment of EGFRm NSCLC patients with central nervous system (CNS) metastases, including LM.

However, considering all relevant progress made in this setting, no study on the relationship between osimertinib treatment and LM development has been reported. Despite significant advances in systemic and local approaches, establishing preventive strategies for LM is more rewarding than administering treatment. Therefore, we conducted a propensity-matched retrospective study to investigate the role of osimertinib therapy for preventing LM in patients with advanced EGFRm NSCLC.

## Patients and methods

### Patients

This single-center, retrospective study was approved by the Institutional Ethics Committee of Second Affiliated Hospital of Nanchang University. Written informed consent was not required due to the retrospective nature of the study. From the clinical records database of our center, we reviewed a total of 1505 patients diagnosed with NSCLC between September 2016 and September 2019 with adequate follow-up data. The eligibility criteria included the following: (1) histological diagnosis of postoperative recurrence or stage IV NSCLC; (2) the presence of activating EGFR mutations; (3) age older than 18 years; and (4) treatment with at least 8 weeks of TKIs as a first-line treatment (erlotinib, gefitinib, afatinib, osimertinib or icotinib). Major exclusion criteria included patients with NSCLC who presented with LM at the time of diagnosis or recurrence. All of the patients with recurrent NSCLC were retrospectively restaged. After the screening procedure, 304 individuals were selected for the analysis. Patients who met the inclusion criteria were divided into two groups: the osimertinib group and the control group. Oligometastatic disease was defined as the presence of ≤5 lesions in 1 to multiple organs at the initiation of TKIs, while polymetastatic disease was defined as > 5 metastatic lesions.

### Treatment and follow-up

Between September 2016 and September 2019, a total of 1505 patients with NSCLC were hospitalized at our center. Three hundred four patients were included in the study population. Among them, 116 patients received osimertinib, and 188 patients did not. In the 1:1 match, patients who received osimertinib therapy were individually matched with one control patient. Variables used for the propensity score matching (PSM) included chemotherapy and antiangiogenic therapy. Baseline laboratory characteristics (hematologic and biochemical profiles) were evaluated every 4 weeks. Magnetic resonance imaging of the brain, chest computed tomography (CT) and upper abdominal CT were performed every 2 months until death or last follow-up. The LM diagnosis at our center was based on the cytological presence of malignant cells in the CSF or typical clinical signs along with positive radiologic findings.

### Statistical analysis

The primary endpoint of the present study was the cumulative incidence of LM, with time to development computed from the date of NSCLC diagnosis or recurrence to the date of LM diagnosis. Patients who did not develop LM were censored on the date of last follow-up or death. The secondary endpoint was OS. Data represent the median (range or 95% confidence interval [CI]) and n (%). Baseline characteristics were compared using Fisher’s exact test for categorical variables and the two-sample t-test or Mann-Whitney U-test for continuous variables, as appropriate. Actuarial rates of freedom for LM development and OS were evaluated by Kaplan-Meier plots and the Cox log-rank test. The cumulative risk for LM was estimated using the cumulative incidence curve. The Cox proportional hazards model was used to run univariate and multivariate analyses to evaluate factors that influenced the occurrence of LM. Variables with *P* values ≤0.10 in the univariate analysis were included in the multivariate analysis. *P*-values < 0.05 were considered to indicate significant differences. The statistical software packages R (http://www.R-project.org, The R Foundation) and Empower Stats (http://www.empowerstats.com, X&Y Solutions, Inc., Boston, MA) were used to analyze all data.

## Results

### Patient characteristics

**Fig. 1:**
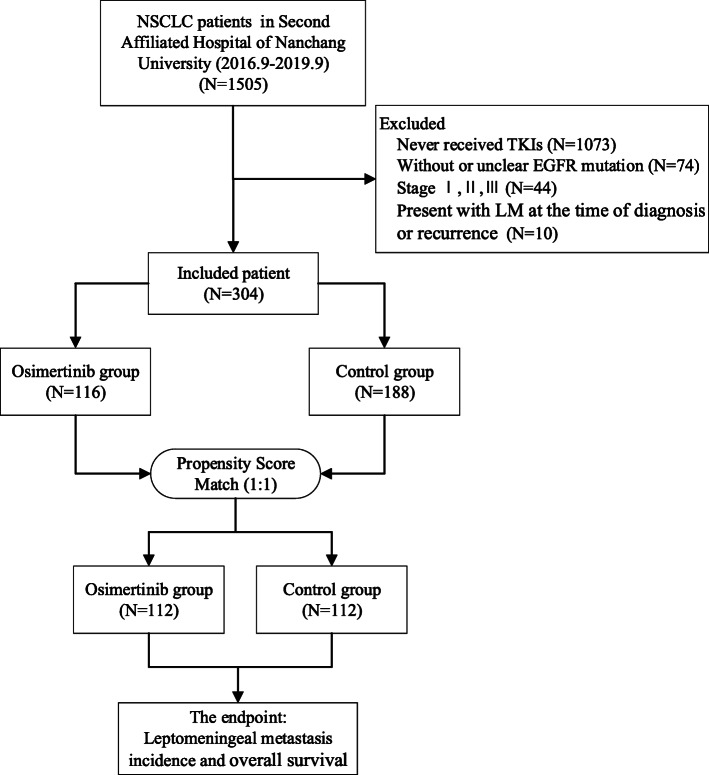
Flow chart of screened patients. NSCLC non-small cell lung cancer, TKIs tyrosine kinase inhibitors, EGFR epidermal growth factor receptor

Between September 2016 and September 2019, a total of 1505 patients with NSCLC were hospitalized at our center. The flow chart of the screened patients is summarized in Fig. 1. Ultimately, 304 patients were included in the study population. Among them, 116 patients were treated with osimertinib (80 mg qd) as first-line treatment or second-line treatment after disease progression on prior TKI therapy during follow-up (osimertinib group), while 188 patients were treated with other TKIs (erlotinib, gefitinib, afatinib or icotinib) as first-line treatment but did not receive osimertinib (control group). For patients in the osimertinib group who received the drug as a second-line treatment (89 of 116 patients), 85/89 (95.5%) had a confirmed T790M mutation. For patients in the control group, 21/188 (11.17%) patients had a confirmed T790M mutation. Patient and tumor characteristics are listed in Table 1. In the osimertinib group, 27 out of 116 (23.28%) patients received osimertinib as first-line treatment, and the remaining 89 patients (76.72%) received osimertinib as second-line treatment after their disease progressed with prior TKI therapy. In the event of disease progression after osimertinib treatment in the osimertinib group or TKI treatment in the control group, the treatment was changed to another systemic therapy. Among the patients (222 of 304) who experienced disease progression, 121 underwent chemotherapy, 92 underwent antiangiogenic therapy, and 15 underwent immunotherapy. Using PSM at a ratio of 1:1, 112 patients remained in each group. After PSM, there were no significant differences in the clinical characteristics between the osimertinib group and the control group.

**Table 1:** Baseline characteristics of the unmatched and matched groups

Characteristic	Before Propensity Score Matching			After Propensity Score Matching		
	Control group	Osimertinib group	***p*** value	Control group	Osimertinib group	***p*** value
**NO. of patients**	188	116		112	112	
**Median age years (range)**	61 (25–87)	60 (33–83)	0.597	58(32–87)	60 (33–83)	0.088
**Sex**			0.171			0.499
Female	100 (53.19%)	71 (61.21%)		62 (55.36%)	67 (59.82%)	
Male	88 (46.81%)	45 (38.79%)		50 (44.64%)	45 (40.18%)	
**Smoking status**			0.182			0.293
Never	131 (69.68%)	89 (76.72%)		78 (69.64%)	85 (75.89%)	
Former or current	57 (30.32%)	27 (23.28%)		34 (30.36%)	27 (24.11%)	
**Histological type**			0.62			0.714
Adenocarcinoma	178 (94.68%)	109 (93.97%)		103 (91.96%)	105 (93.75%)	
Adenosquamous carcinoma	4 (2.13%)	3 (2.59%)		4 (3.57%)	3 (2.68%)	
Not otherwise specified	4 (2.13%)	1 (0.86%)		3 (2.68%)	1 (0.89%)	
Squamous carcinoma	2 (1.06%)	3 (2.59%)		2 (1.79%)	3 (2.68%)	
**EGFR mutations**			0.208			0.458
Exon19 deletion	96 (51.06%)	71 (61.21%)		60 (53.57%)	69 (61.61%)	
L858R mutation	84 (44.68%)	42 (36.21%)		48 (42.86%)	40 (35.71%)	
Others	8 (4.26%)	3 (2.59%)		4 (3.57%)	3 (2.68%)	
**T**			0.363			0.777
0	13 (6.91%)	10 (8.62%)		7 (6.25%)	10 (8.93%)	
1	44 (23.40%)	18 (15.52%)		16 (14.29%)	18 (16.07%)	
2	85 (45.21%)	63 (54.31%)		59 (52.68%)	60 (53.57%)	
3	22 (11.70%)	10 (8.62%)		14 (12.50%)	9 (8.04%)	
4	24 (12.77%)	15 (12.93%)		16 (14.29%)	15 (13.39%)	
**N**			0.408			0.337
0	42 (22.34%)	30 (25.86%)		25 (22.32%)	30 (26.79%)	
1	9 (4.79%)	10 (8.62%)		7 (6.25%)	10 (8.93%)	
2	114 (60.64%)	61 (52.59%)		71 (63.39%)	58 (51.79%)	
3	23 (12.23%)	15 (12.93%)		9 (8.04%)	14 (12.50%)	
**M**			0.63			0.704
1a	32 (17.02%)	15 (12.93%)		18 (16.07%)	14 (12.50%)	
1b	12 (6.38%)	8 (6.90%)		9 (8.04%)	8 (7.14%)	
1c	144 (76.60%)	93 (80.17%)		85 (75.89%)	90 (80.36%)	
**Clinical stage**			0.601			0.485
IVa	32 (17.02%)	15 (12.93%)		20 (17.86%)	14 (12.50%)	
IVb	135 (71.81%)	86 (74.14%)		80 (71.43%)	83 (74.11%)	
Recurrence	21 (11.17%)	15 (12.93%)		12 (10.71%)	15 (13.39%)	
**Brain metastasis**			0.224			0.414
No	105 (55.85%)	73 (62.93%)		64 (57.14%)	70 (62.50%)	
Yes	83 (44.15%)	43 (37.07%)		48 (42.86%)	42 (37.50%)	
**Bone metastasis**			0.718			0.789
No	90 (47.87%)	58 (50.00%)		59 (52.68%)	57 (50.89%)	
Yes	98 (52.13%)	58 (50.00%)		53 (47.32%)	55 (49.11%)	
**Liver metastasis**			0.824			1
No	162 (86.17%)	101 (87.07%)		98 (87.50%)	98 (87.50%)	
Yes	26 (13.83%)	15 (12.93%)		14 (12.50%)	14 (12.50%)	
**Adrenal metastasis**			0.349			0.299
No	173 (92.02%)	110 (94.83%)		102 (91.07%)	106 (94.64%)	
Yes	15 (7.98%)	6 (5.17%)		10 (8.93%)	6 (5.36%)	
**Metastatic status**			0.428			0.277
Oligometastasis	114 (60.64%)	65 (56.03%)		70 (62.50%)	62 (55.36%)	
Polymetastasis	74 (39.36%)	51 (43.97%)		42 (37.50%)	50 (44.64%)	
**First-line EGFR-TKIs**			< 0.001*			< 0.001*
Gefitnib	129 (68.62%)	55 (47.41%)		74 (66.07%)	54 (48.21%)	
Erlotinib	14 (7.45%)	12 (10.34%)		13 (11.61%)	12 (10.71%)	
Osimertinib	0 (0.00%)	27 (23.28%)		0 (0.00%)	27 (24.11%)	
Icotinib	35 (18.62%)	20 (17.24%)		21 (18.75%)	18 (16.07%)	
Afatinib	10 (5.32%)	2 (1.72%)		4 (3.57%)	1 (0.89%)	
**T790M**			< 0.001*			< 0.001*
No or unknow	167 (88.8%)	31 (26.7%)		100 (89.3%)	29 (25.9%)	
Yes	21 (11.2%)	85 (73.3%)		12 (10.7%)	83 (74.1%)	
**Chemotherapy**			0.033*			0.109
No	122 (64.89%)	61 (52.59%)		49 (43.75%)	61 (54.46%)	
Yes	66 (35.11%)	55 (47.41%)		63 (56.25%)	51 (45.54%)	
**Antiangiogenic therapy**			0.022*			0.081
No	140 (74.47%)	72 (62.07%)		84 (75.00%)	72 (64.29%)	
Yes	48 (25.53%)	44 (37.93%)		28 (25.00%)	40 (35.71%)	
**Immunotherapy**			0.347			0.061
No	177 (94.15%)	112 (96.55%)		101 (90.18%)	108 (96.43%)	
Yes	11 (5.85%)	4 (3.45%)		11 (9.82%)	4 (3.57%)	
**Chest radiotherapy**			0.186			0.088
No	140 (74.47%)	94 (81.03%)		79 (70.54%)	90 (80.36%)	
Yes	48 (25.53%)	22 (18.97%)		33 (29.46%)	22 (19.64%)	
**WBRT**			0.425			0.068
No	128 (68.09%)	84 (72.41%)		68 (60.71%)	81 (72.32%)	
Yes	60 (31.91%)	32 (27.59%)		44 (39.29%)	31 (27.68%)	
**Number of lines of osimertinib therapy**			1			1
First-line	0 (%)	27 (23.28%)		0 (%)	27 (24.11%)	
Second-line	0 (%)	89 (76.72%)		0 (%)	85 (75.89%)	
**Median FU (95% CI)**	18.3 (16.3–19.6)	24.2 (21.2–26.5)	< 0.001*	23.5 (19.3–24.0)	25.5 (21.4–26.9)	0.087

*EGFR* epidermal growth factor receptor, *TKIs* tyrosine kinase inhibitors, *WBRT* whole brain radiation therapy, *FU* follow-up time, *CI* confidence interval

* *P*<0.05 was considered significant. Data represent the median (range or 95% CI) and n (%)

### Incidence rates of LM

**Fig. 2:**
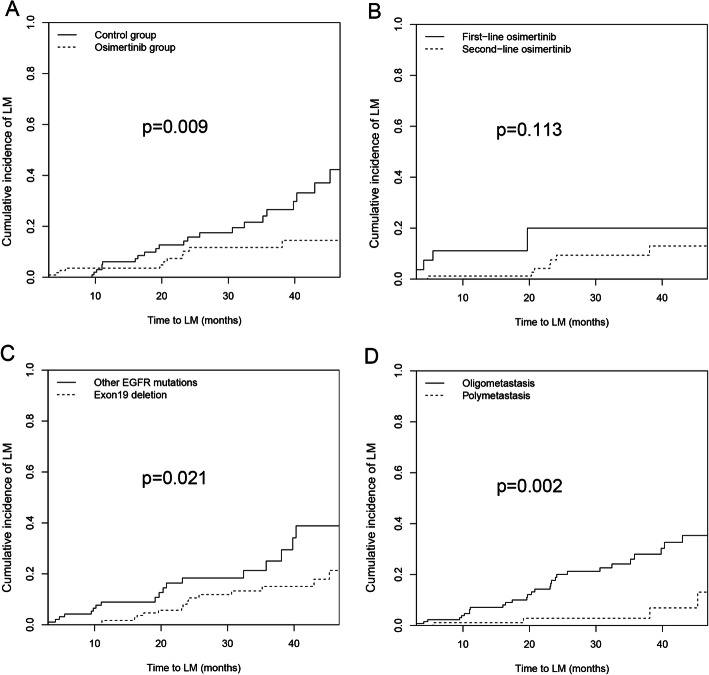
Cumulative incidence of leptomeningeal metastasis in patients after propensity score matching. **a** Osimertinib group vs Control group; **b** First-line osimertinib vs Second-line osimertinib; **c** Exon 19 deletion vs Other EGFR mutations; **d** Polymetastasis vs Oligometastasis. LM leptomeningeal metastasis, EGFR epidermal growth factor receptor

The median follow-up time for the osimertinib group was 25.5 months (95% CI 21.4–26.9 months), which was comparable to the median follow-up time of the control group (23.5 months 95% CI 19.3–24.0 months). In the osimertinib group, LM developed in 11 patients (9.82%), and the median time to LM development was 20.3 months (95% CI 7.4–21.4 months), with 1-year and 2-year cumulative incidence rates of LM of 3.57 and 8.03%, respectively. Findings from the control group were different*,* with LM developing in 24 patients (21.42%). The median time to LM development was 23.6 months (95% CI 17.9–28.2 months), with 1-year and 2-year cumulative incidence rates of LM of 5.36 and 11.60%, respectively. There was a significant difference in the cumulative risk for LM between the two groups (hazard ratio [HR] 0.38, 95% CI 0.19–0.79, *p* = 0.009) (Fig. 2a). Our results suggest that the risk of LM in the osimertinib group was significantly lower than that in the control group. Subgroup analysis of osimertinib as a first-line vs. second-line therapy revealed no significant difference in the cumulative incidence rates of LM (4/27 [14.81%] vs. 7/85 [8.24%]) when comparing first-line osimertinib therapy to second-line osimertinib therapy. (HR 0.36, 95% CI 0.11–1.27, *p* = 0.113) (Fig. 2b).

**Fig. 3:**
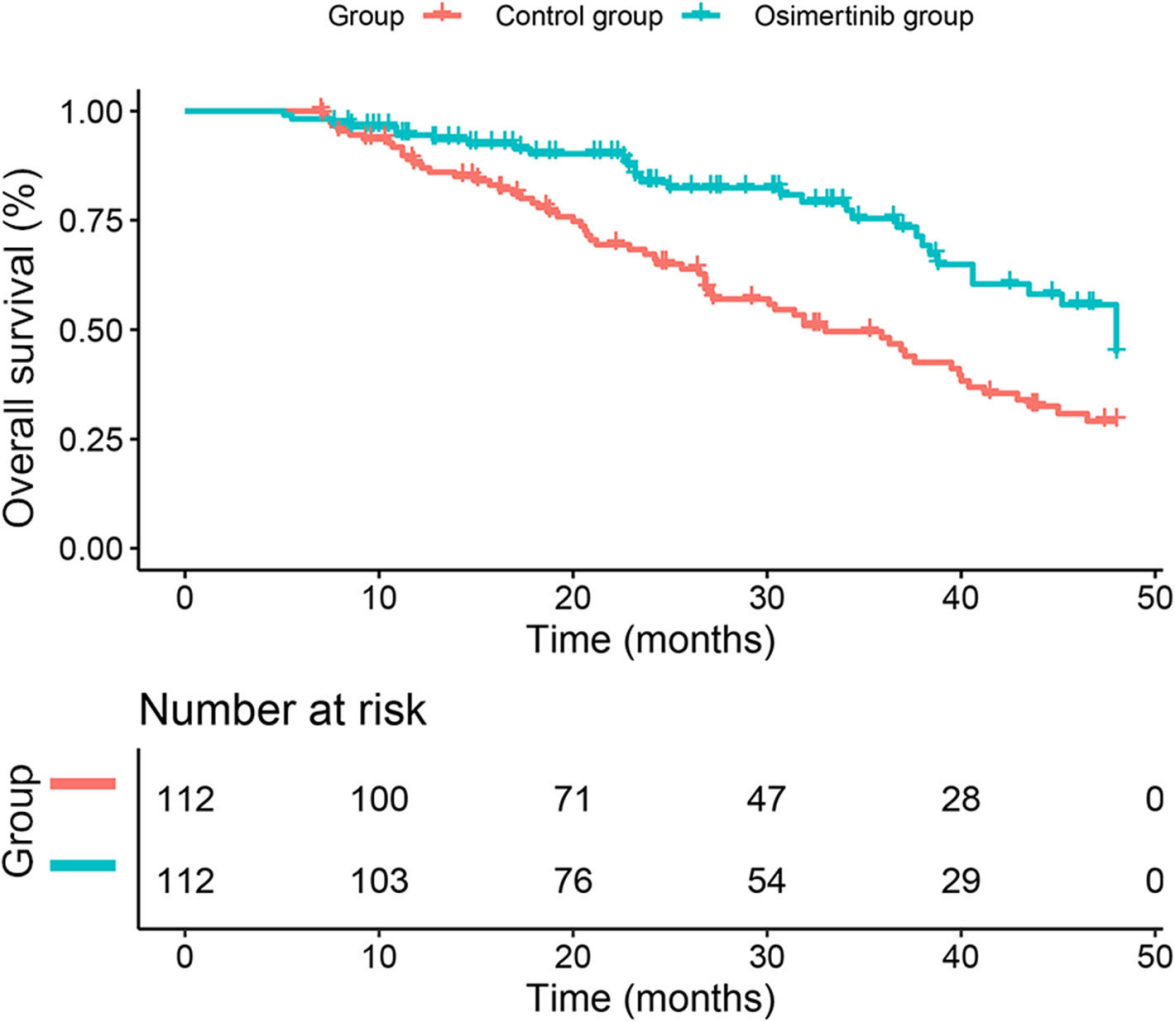
Kaplan-Meier curves for median overall survival. LM leptomeningeal metastasis

At present, the available OS rate data are too immature (42.41% maturity) for statistical analysis. At the endpoint of the study, 33 deaths (29.46%) had occurred in the osimertinib group and 62 (55.36%) in the control group (Fig. 3).

### Univariate and multivariate analyses of risk for LM

Univariate analysis revealed that the use of osimertinib, liver metastasis, EGFR mutations (Fig. 2c), and metastatic status (Fig. 2d) were potential prognostic factors for LM (Table 2). Among these, the use of osimertinib (Yes vs No) (HR 0.33, 95% CI 0.11-0.96, *p* = 0.042), EGFR mutations (19del vs Others) (HR 0.34, 95% CI 0.16-0.72, *p* = 0.004) and metastatic status (Polymetastasis vs Oligometastasis) (HR 0.13, 95% CI 0.04–0.39, *p* < 0.001) were independent prognostic factors for LM in the multivariate analysis (Table 2).

**Table 2:** Univariate and multivariate analysis for leptomeningeal metastasis incidence

**Univariate analysis (*N*** **= 224)**				
**Variable name**	**HR**	**95% CI for HR**		***p*-value**
Age (≥65 vs <65)	0.71	0.34	1.48	0.357
Gender (Male vs female)	1.69	0.87	3.29	0.120
Smoking status (Former or current vs Never)	1.91	0.97	3.75	0.062
Histology (Adenocarcinoma vs Others)	2.14	0.29	15.65	0.453
EGFR mutations (Exon19 deletion vs Others)	0.46	0.23	0.89	0.021*
T (T2–4 vs T0–1)	0.60	0.30	1.20	0.151
N (N1–3 vs N0)	1.14	0.53	2.43	0.740
M (M1c vs Others)	1.19	0.59	2.40	0.621
Clinical stage (Recurrence vs IV)	2.07	0.97	4.43	0.060
Brain metastasis (Yes vs No)	1.32	0.67	2.59	0.416
Bone metastasis (Yes vs No)	1.29	0.66	2.50	0.455
Liver metastasis (Yes vs No)	2.44	1.06	5.61	0.035*
Adrenal metastasis (Yes vs No)	1.30	0.31	5.44	0.722
Metastatic status (Polymetastasis vs Oligometastasis)	0.19	0.07	0.55	0.002*
First-line EGFR TKIs				
Gefitnib	Reference			
Erlotinib	0.46	0.14	1.55	0.213
Osimertinib	1.08	0.37	3.13	0.887
Icotinib	0.63	0.22	1.82	0.392
Afatinib	1.93	0.26	14.45	0.524
T790M (Yes vs No)	0.56	0.29	1.11	0.099
Chemotherapy (Yes vs No)	0.79	0.40	1.53	0.475
Antiangiogenic therapy (Yes vs No)	1.04	0.52	2.08	0.906
Immunotherapy (Yes vs No)	1.39	0.42	4.54	0.587
Chest radiotherapy (Yes vs No)	0.46	0.19	1.11	0.084
WBRT (Yes vs No)	1.43	0.73	2.78	0.296
Use of Osimertinib (Yes vs No)	0.38	0.19	0.79	0.009*
**Multivariate analysis (*N*** **= 224)**				
**Variable name**	**HR**	**95% CI for HR**		***p*-value**
EGFR mutation (Exon19 deletion vs Others)	0.34	0.16	0.72	0.004*
T790M (Yes vs No)	1.50	0.53	4.23	0.446
Metastatic status (Polymetastasis vs Oligometastasis)	0.13	0.04	0.39	0.000*
Use of Osimertinib (Yes vs No)	0.33	0.11	0.96	0.042*

Adjust model adjust for multivariate analysis: Smoking status, EGFR mutations, Clinical stage, Liver metastasis, Metastatic status, T790M, Chest radiotherapy, Use of Osimertinib

*HR* hazard ratio, *CI* confidence interval, *EGFR* epidermal growth factor receptor, *TKIs* tyrosine kinase inhibitors, *WBRT* whole brain radiation therapy

* *P*<0.05 was considered significant

## Discussion

LM is a serious complication of advanced NSCLC that causes severe damage to neurocognitive functions and affects patients’ quality of life and prognosis [1, 9]. There is no standard treatment for this disease. Several therapeutic options, including intrathecal chemotherapy and radiotherapy, have been applied to manage LM [1, 15–17]. However, these treatments have limited antitumor activity and/or are associated with significant toxicity and complications [1, 15–18]. More recently, the use of EGFR-TKIs, particularly osimertinib, has improved systemic disease control and prolonged survival for subgroups of patients with EGFRm NSCLC and LM [11, 12]. However, the use of osimertinib for the prevention of LM in NSCLC patients with EGFRm remains unclear.

Osimertinib, a third-generation TKI designed to target both EGFR sensitizing mutations and T790M, was approved for the first-line treatment of patients with EGFRm advanced NSCLC and for the treatment of EGFR T790M mutation-positive NSCLC patients with progressive disease after prior EGFR TKI therapy [19, 20]. Our results indicated the incidence of LM was significantly reduced by osimertinib. LM developed in 11 patients (9.82%) in the osimertinib group and in 24 patients (21.42%) in the control group (*p* = 0.009). The cumulative risks for LM in the osimertinib group at 1 year and 2 years were 3.57 and 8.03%, respectively, whereas the rates were 5.36 and 11.6%, respectively, in the control group. In the multivariate analysis, a Cox regression model also showed that osimertinib was an independent, statistically significant predictor for determining the risk for LM (HR 0.33, 95% CI 0.11–0.96, *p* = 0.042). This suggests that osimertinib can prevent the occurrence of LM in patients with advanced EGFRm NSCLC.

Osimertinib significantly prolonged the progression-free survival in treatment-naïve patients with EGFRm advanced NSCLC and in patients with EGFR T790M NSCLC after disease progression with prior EGFR-TKIs treatment [21–23]. Osimertinib also demonstrated superior CNS efficacy in T790M-positive NSCLC [24]. Despite these advances, various barriers still often result the refusal of osimertinib treatment in clinical practice [25, 26]. In the present study, only 27/304 (8.88%) patients received osimertinib as the first-line treatment. In the control group, 21/188 patients with a confirmed T790M mutation did not receive osimertinib as second-line treatment. In the osimertinib group, subgroup analysis showed no significant difference in the cumulative incidence rates of LM when comparing osimertinib as a first-line vs second-line therapy. Given the increasing incidence of LM in recent years and the potential preventive role of osimertinib, osimertinib treatment should be recommended for patient populations likely to benefit from it.

The number of T790M confirmed cases in control group is only 12 after PSM. Patients with acquired T790M mutation had a prolonged survival compared to patients with negative T790M [27]. Thus, it is theoretically possible that the risk of LM in patients with T790M should be higher than that in patients with negative T790M throughout the course of the cancer disease. Whereas during the comparable follow up period, our results suggest that the risk of LM in the osimertinib group (83/112 patients with T790M mutation) was significantly lower than that in the control group (only 12/112 patients with T790M mutation). Furthermore, the progression pattern analysis of T790M positive and negative patients has confirmed that CNS progression is independent of acquired T790M mutation [27]. Therefore, it might be that osimertinib but not T790M played a role in reducing the incidence of LM in patients with advanced NSCLC harboring EGFR mutations.

Previous studies showed that LM occurs in 9.0 to 9.4% of patients with EGFR mutations [3, 4]; however, the incidence was 15.6% (35 out of 224) in our study. The higher incidence of LM observed in our study may be because the outpatients with EGFRm advanced NSCLC were not enrolled. Additionally, we found that patients harboring 19del were less likely to experience LM than those harboring other EGFR mutations (*p* = 0.021). This finding was consistent with that of a prior study that revealed that 19del potentially predicts a lower risk for LM than L858R [28]. Our data also indicate that the incidence of LM was higher in patients with oligometastatic disease than in those with polymetastatic disease (*p* = 0.002). One probable reason is that patients with oligometastases may survive longer than those with polymetastases [29]. As patient survival lengthens, the risk for LM increases.

The goal of radiotherapy for LM is to palliate symptoms and to improve CSF flow. There were conflicting findings on whether whole brain radiation therapy (WBRT) improved survival for patients with NSCLC and LM [1, 9, 10, 16, 30]. A retrospective review of 125 patients with NSCLC and LM reported that survival was not improved by WBRT [16]. In contrast, another retrospective study showed that patients who underwent WBRT for LM survived longer (8.4 months vs 1.8 months, *p* < 0.001) [10]. Craniospinal irradiation (CSI) for the treatment of LM is rarely used because of its substantial toxicity and complications [15, 18]. In a retrospective study of patients who received CSI, 32% developed grade III myelosuppression [18]. A meta-analysis of seven studies including 2114 patients with small cell lung cancer showed that prophylactic cranial irradiation has a significant effect on decreasing BM [31]. In our study, 90 patients had known BM. Among these patients, 75 (83.33%) patients underwent WBRT, which did not appear to play a preventive role against LM (HR 1.43, 95% CI 0.73–2.78, *p* = 0.296). The therapeutic and prophylactic effects of radiotherapy (WBRT, CSI and radiosurgery) for LM need to be studied further.

Although this study provides meaningful data, we acknowledge several limitations. First, this study was based on a retrospective analysis performed at a single center with potential hidden biases. The adoption of PSM balances baseline patient characteristics between groups, and multivariate regression was used to minimize some weaknesses of a retrospective study. The second limitation is the low number of patients treated with osimertinib as the first-line treatment; osimertinib was not covered by health insurance before March 2021 as per the local government policy.

## Conclusions

In conclusion, we observed that osimertinib can effectively decrease the incidence of LM in patients with advanced common EGFR mutations NSCLC in clinical practice. Interestingly, patients harboring deletion mutations of EGFR exon 19 were less likely to experience LM than those with other EGFR mutations. Therefore, osimertinib might be a suitable option for specific patient populations with advanced common EGFR mutations NSCLC. However, additional studies are needed to confirm these findings.

## Data Availability

The datasets used and/or analyzed during the current study are available from the corresponding author on reasonable request.

## Abbreviations

LM: Leptomeningeal metastasis
NSCLC: Non-small cell lung cancer
EGFR: Epidermal growth factor receptor
TKIs: Tyrosine kinase inhibitors
PSM: Propensity score matching
OS: Overall survival
HR: Hazard ratio
CI: Confidence interval
BBB: Blood-brain barrier
CSF: Cerebrospinal fluid
BM: Brain metastasis
EGFRm: EGFR-mutated
CNS: Central nervous system
CT: Computed tomography
WBRT: Whole brain radiation therapy
CSI: Craniospinal irradiation
